# The *Rac1* Promoter as a Molecular Nexus for Antihypertensive Therapy Resistance and Enhanced Malaria Susceptibility

**DOI:** 10.1155/ijhy/8287621

**Published:** 2026-04-21

**Authors:** Selassie Louis Ameke, Kwadwo Fosu, Lucas Amenga-Etego, Kwabena Amofa Nketia Sarpong, Samuel Kojo Kwofie

**Affiliations:** ^1^ West African Centre for Cell Biology of Infectious Pathogens, University of Ghana, Accra, Ghana, ug.edu.gh; ^2^ Department of Biochemistry, Cell and Molecular Biology, University of Ghana, Accra, Ghana, ug.edu.gh; ^3^ Department of Medical Laboratory, Ho Municipal Hospital of Ghana Health Service, Ho, Ghana; ^4^ Department of Biomedical Engineering, University of Ghana, Accra, Ghana, ug.edu.gh; ^5^ Institute of Applied Science and Technology, University of Ghana, Legon, Accra, Ghana, ug.edu.gh

**Keywords:** CpG island, erythrocyte, hypertension, in silico analysis, malaria, mineralocorticoid receptor, promoter, *Rac1*, Sub-Saharan Africa, therapy resistance

## Abstract

**Background:**

In Sub‐Saharan Africa, the escalating burden of hypertension converges with persistent malaria endemicity, creating a complex clinical challenge marked by increasing rates of resistance to first‐line antihypertensive therapies, particularly angiotensin‐converting enzyme inhibitors (ACEIs) and Angiotensin II receptor blockers (ARBs). The molecular mechanisms underpinning this therapeutic failure remain elusive. *Rac1*, a Rho GTPase regulating both cardiovascular function and erythrocyte biology, presents a compelling molecular link between these comorbidities, yet its regulatory architecture in this context is uncharacterized.

**Methods:**

We conducted a comprehensive *in silico* analysis of the human *Rac1* promoter region (−2000 to +500 bp relative to the transcription start site) using biomaRt, BSgenome, and the JASPAR2022 database, anchored to the GRCh38/hg38 reference genome. African ancestry variants from resources such as gnomAD were considered to enhance population‐specific relevance. Transcription factor binding sites were predicted using position weight matrices with an 80% relative score threshold to balance sensitivity and specificity. CpG island analysis was performed, including calculation of the observed/expected (o/e) ratio.

**Results:**

The 2501 bp *Rac1* promoter is notably GC‐rich (57.34%) and contains 164 CpG sites with an o/e ratio of 0.75, defining a canonical CpG island. Analysis revealed a complex regulatory landscape featuring binding motifs for the mineralocorticoid receptor (MR), suggesting a potential pathway for bypassing RAAS blockade and contributing to ACEI/ARB resistance. Binding sites for hypoxia‐inducible factors, inflammatory mediators, and the erythroid‐specific factor GATA‐1 were also identified. Spatial analysis showed nonrandom clustering of these elements, suggesting integrated response capabilities.

**Conclusion:**

The regulatory architecture of the *Rac1* promoter suggests a potential molecular basis for MR‐mediated resistance to ACEI/ARB therapies while simultaneously providing a predictive link to enhanced malaria susceptibility through erythrocyte remodeling pathways. These findings offer a novel framework for understanding treatment‐resistant hypertension in malaria‐endemic regions and identify the *Rac1* promoter as a candidate nexus for developing dual‐disease therapeutic strategies tailored to high‐burden populations.

## 1. Introduction

The convergence of noncommunicable and infectious diseases represents an emerging global health challenge, particularly in regions where hypertension and malaria coexist as significant public health burdens. This convergence is particularly acute in Sub‐Saharan Africa, where population‐specific factors such as a high genetic diversity shaped by historical evolutionary pressures, including endemic malaria, and distinct environmental exposures may influence disease pathogenesis and therapeutic response.

The management of hypertensive patients in malaria‐endemic areas presents unique clinical challenges, with growing evidence suggesting altered efficacy of conventional antihypertensive therapies [1, 2]. Renin−angiotensin−aldosterone system (RAAS) inhibitors, while demonstrating robust efficacy across diverse populations, exhibit variable therapeutic responses in malaria‐endemic regions [3, 4] and suggest potential pathogen‐mediated modifications of cardiovascular regulatory pathways that warrant mechanistic investigation.

The *Rac1* protein, a pivotal member of the Rho GTPase family, functions as a molecular switch governing diverse cellular processes including cytoskeletal dynamics [5], reactive oxygen species production [6], and transcriptional regulation [7]. Emerging evidence implicates *Rac1* in both cardiovascular pathophysiology [8, 9] and host−pathogen interactions [10], positioning it as a potential molecular link between these clinically intersecting conditions. Particularly relevant is the demonstrated role of the RAAS pathway in providing innate defense against *Plasmodium falciparum* invasion of erythrocytes [10, 11], raising concerns that RAAS inhibitor use may inadvertently compromise this protective mechanism and enhance malaria susceptibility in endemic populations. This defense is hypothesized to involve RAAS‐mediated maintenance of endothelial integrity and modulation of inflammatory responses, which may create a less permissive environment for parasite sequestration and proliferation. Consequently, pharmacological RAAS blockade could compromise these protective mechanisms, potentially increasing host vulnerability to malaria infection.

The molecular mechanisms underlying these clinical observations remain poorly characterized. We hypothesize that *Rac1* protein promotes antihypertensive therapy resistance through mineralocorticoid receptor (MR)−mediated signaling that bypasses conventional RAAS blockade while simultaneously enhancing malaria susceptibility via cytoskeletal remodeling of erythrocytes that facilitates parasite invasion.

This study aims to comprehensively characterize the regulatory architecture of the *Rac1* promoter to elucidate the molecular mechanisms underlying these interconnected clinical phenomena. Through detailed analysis of transcription factor binding sites (TFBSs), epigenetic regulatory elements, and structural features of the *Rac1* promoter region, we seek to provide mechanistic insights that could inform novel therapeutic strategies for managing this clinically significant comorbidity in endemic regions. Our findings may ultimately contribute to the development of targeted interventions that simultaneously address both conditions while accounting for population‐specific genetic and epigenetic factors.

## 2. Methods

### 2.1. Promoter Sequence Retrieval and Analysis

The human *Rac1* promoter sequence was retrieved from the GRCh38/hg38 reference genome using two complementary approaches to ensure accuracy. Primary sequence extraction was performed using the biomaRt package (V2.58.0) [12] in R, with cross‐verification through BSgenome.Hsapiens.UCSC.hg38 (V1.4.5) [13]. The analyzed region spanned 2501 base pairs, encompassing 2000 bp upstream to 500 bp downstream [14, 15] of the primary transcription start site (TSS) (chr7:6, 374, 527), corresponding to genomic coordinates chr7:6, 372, 527–6,375,027. This region was selected to capture both core promoter elements and potential distal regulatory sequences based on established promoter architecture studies.

### 2.2. TFBS Prediction

Comprehensive TFBS analysis was conducted using JASPAR2022 (V0.99.10) [16], a curated database of transcription factor binding preferences. Position weight matrices for vertebrate transcription factors were employed through the TFBSTools package (V1.40.0) [17] in Bioconductor. To ensure high‐confidence predictions, we applied a conservative threshold of 80% relative score compared to the matrix maximum. This cutoff is stringent to capture a broader, hypothesis‐generating set of potential regulatory interactions while significantly reducing false positive identifications and maintaining sensitivity for biologically relevant sites [18, 19]. Only predictions meeting this stringent cutoff across both DNA strands were considered for subsequent analysis.

### 2.3. Epigenetic Feature Characterization

The CpG island analysis was performed using the Biostrings package (V2.70.2) [20, 21] for efficient pattern matching and sequence manipulation. The CpG dinucleotides were identified through exact pattern matching, with density analysis conducted using 100 bp sliding windows advanced in 50 bp increments to provide overlapping coverage and smooth density estimates. This approach allowed for precise mapping of CpG‐rich regions while maintaining resolution of localized clustering patterns.

### 2.4. Statistical and Spatial Analysis

The spatial organization of predicted TFBSs within the 2501 bp *Rac1* promoter was analyzed using custom R scripts (R Foundation for Statistical Computing, V4.3.1) to assess density, clustering, and positional enrichment. The following algorithmic pipeline was implemented:

1. TFBS density calculation: To visualize the distribution of regulatory elements, a sliding window analysis was performed. Density was computed in 100 bp windows incremented by 50 bp across the entire promoter sequence. For each window *i*, we calculated the density as
(1) density i=NiL×100,
 where *N*_*i*_ is the number of TFBS whose midpoint falls within window *i*, and *L* is the window length (100 bp). This yields a density value representing the percentage of the window occupied by a TFBS midpoint.
2. Clustering detection algorithm: Nonrandom spatial clustering of TFBS was evaluated using a two‐step approach.
 Distance‐based identification: All pairwise distances between TFBS midpoints were calculated. A spatial cluster was preliminarily defined as a group of three or more TFBS where the maximum interelement distance was ≤ 200 bp, based on established conventions for proximal regulatory modules in promoter architecture.
 Statistical significance testing: The significance of observed clustering was assessed by comparing the observed mean inter‐TFBS distance to a null distribution. We generated 1000 random permutations, distributing the same total number of TFBS uniformly across the promoter region while preserving its length. A *Z*‐score was calculated to quantify deviation from randomness as
(2) Z=Dobserved−µrandomδrandom,
 where *D*_(observed)_ is the mean inter‐TFBS distance in the experimental data, and µ_(random)_ and δ_(random)_ are the mean and standard deviation of the mean distances from the permuted datasets.
 A *Z*‐score ≤ −2.0 (corresponding to a *p* value < 0.05) was considered indicative of significant clustering.
3. Positional enrichment analysis: To determine if specific functional categories of TFBS (e.g., inflammatory mediators vs. erythroid factors) were enriched in particular promoter subregions (e.g., proximal vs. distal), a contingency table analysis was performed. The promoter was divided into logical subregions based on initial density plots. For each TFBS category and subregion, Fisher’s exact test (two‐tailed) was used to compare the observed frequency against the frequency expected under a uniform distribution of all TFBS.
4. Visualization: All spatial distribution data were visualized using the “ggplot2” package (V3.4.0). The primary plot displayed regulatory element density (*y*‐axis) against genomic position relative to the TSS (Position 0) (*x*‐axis). Peak regions, defined as contiguous windows where density exceeded the 75th percentile of all calculated densities, were annotated directly on the plot using the “annotate()” function.

## 3. Results

### 3.1. *Rac1* Promoter Architecture and Sequence Features

**FIGURE 1:**
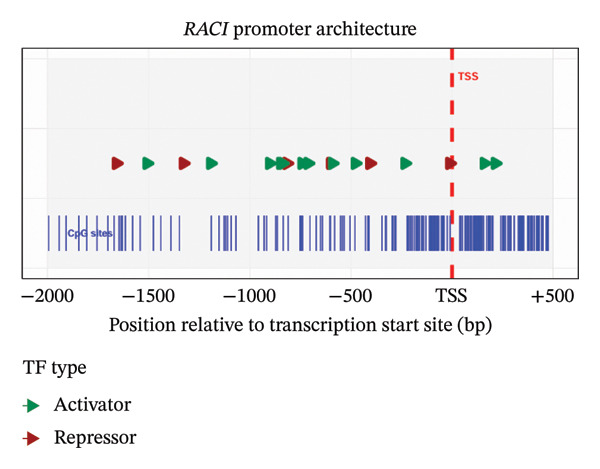
The spatial architecture of the *Rac1* gene regulatory elements.

Our analysis defined the human *Rac1* core promoter as a 2501 bp region on Chromosome 7 (6, 372, 527–6, 375, 027 bp). This region displayed a significantly GC‐rich composition of 57.34%, substantially exceeding the genomic average (41.00%) and the typical composition of housekeeping genes (52.10%) (Table 1). We identified multiple TSSs distributed across the promoter, suggesting a mechanism for alternative promoter usage and transcript diversity that could enable context‐specific expression regulation. This complex architecture is indicative of a gene under sophisticated multilevel control (Figure 1), consistent with its involvement in diverse physiological and pathological processes, while the quantitative statistical comparisons are summarized in Table 1.

**TABLE 1:** The quantitative statistics features of the *Rac1* promoter.

Feature	Region	Value	*Z*_score	*p*_value	Significance
Chromosome	6, 372, 527–6, 375, 027 bp	7	………….	………….	…………….
GC content	*Rac1* promoter	57.34	3.21	< 0.001	∗∗∗
GC content	Housekeeping gene	52.10	1.89	0.03	∗
GC content	Tissue‐specific	48.30	1.25	0.21	NS
GC content	Random intergenic	39.80	−0.23	0.82	NS
GC content	Genomic average	41.00	0.00	1.00	Ref
CpG density	*Rac1* promoter	10.30	2.89	< 0.001	∗∗∗
CpG density	Housekeeping gene	8.20	1.65	0.04	∗
CpG density	Tissue‐specific	6.50	0.89	0.37	NS
CpG density	Random intergenic	2.10	−1.32	0.03	∗
CpG density	Genomic average	4.50	0.00	1.00	Ref
CpG O/E ratio	*Rac1* promoter	0.75	2.45	< 0.01	∗∗
CpG O/E ratio	Housekeeping gene	0.68	1.56	0.03	∗
CpG O/E ratio	Tissue‐specific	0.59	0.87	0.39	NS
CpG O/E ratio	Random intergenic	0.32	−1.89	< 0.01	∗∗
CpG O/E ratio	Genomic average	0.48	0.00	1.00	Ref

*Note:* Statistical significance is determined relative to the genomic average (reference). Asterisks indicate significance levels: *p* < 0.05 (^∗^), *p* < 0.01 (^∗∗^), *p* < 0.001 (^∗∗∗^), and NS indicates not significant (*p* ≥ 0.05). “Ref” denotes the reference group.

### 3.2. Transcription Factor Binding Landscape

**FIGURE 2:**
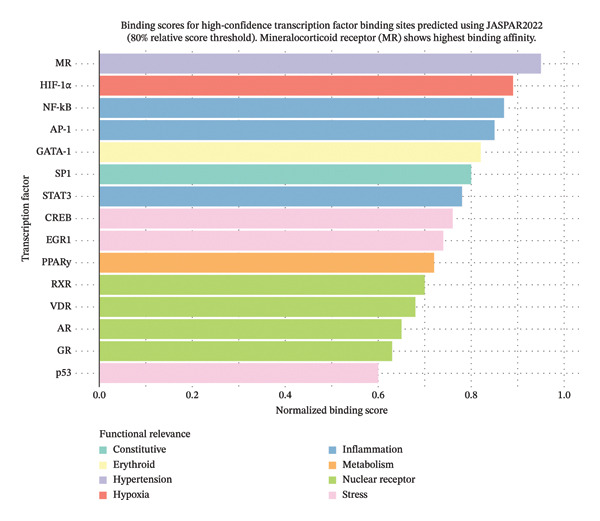
Transcription factor binding score distribution for *Rac1* promoter.

Computational prediction of TFBSs revealed a rich regulatory landscape within the *Rac1* promoter (Figure 2). Notably, we identified multiple putative MR response elements, providing molecular support for direct MR‐mediated *Rac1* regulation that could facilitate bypass of conventional RAAS blockade. The promoter also contains hypoxia‐responsive elements, including binding sites for *HIF-1α* and other oxygen‐sensitive transcription factors, indicating *Rac1* responsiveness to hypoxic conditions relevant to both cardiovascular pathophysiology and malaria pathogenesis. Furthermore, we detected binding sites for inflammatory mediators, including NF‐κB and AP‐1, suggesting *Rac1* regulation through inflammatory signaling pathways that may link hypertension‐associated inflammation with malaria immune responses. Crucially, the presence of erythroid‐specific factors such as *GATA-1* binding sites suggests potential *Rac1* expression modulation in erythrocyte precursors, which may be relevant to malaria parasite invasion mechanisms.

### 3.3. Epigenetic Regulatory Potential

**FIGURE 3:**
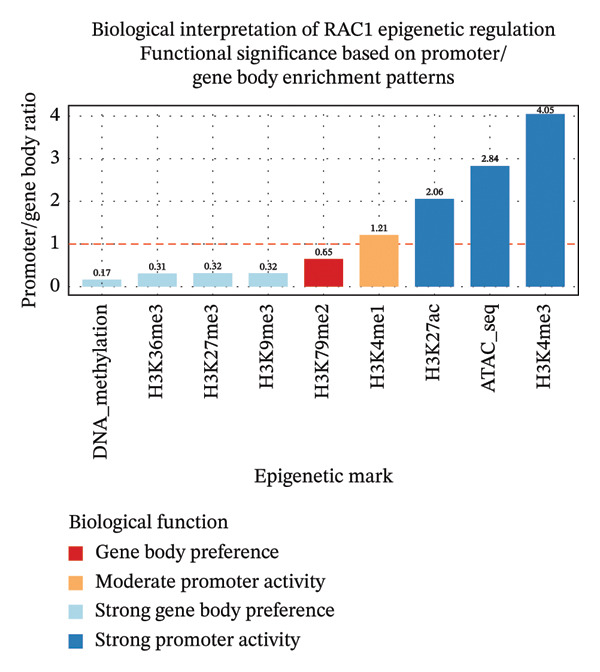
DNA‐methylation modifications at CpG sites and histone modifications around the *Rac1* promoter.

Our analysis identified 164 CpG sites within the promoter region, revealing substantial potential for epigenetic regulation in the form of DNA‐methylation and histone (H3) modification (Figure 3). The CpG distribution demonstrated regional clustering with particular density in the −800 to −200 bp region relative to the TSS. This epigenetic architecture suggests three key regulatory implications: first, tissue‐specific regulation through differential methylation could modulate *Rac1* expression in cardiovascular tissues versus erythroid cells; second, the CpG‐rich regions may function as environmental sensors responsive to factors including oxidative stress and inflammatory mediators; third, variable methylation patterns could underlie observed population differences in both antihypertensive therapeutic response and malaria susceptibility, providing a potential molecular basis for geographical heterogeneity in disease presentation. The notably high H3K4me3 promoter‐to‐gene body ratio suggests intense, focused promoter activity, potentially indicative of a transcriptionally permissive state during acute inflammatory conditions such as active malaria infection. In contrast, under chronic hypertensive conditions, this pattern may reflect sustained but dysregulated promoter engagement contributing to pathological *Rac1* expression.

### 3.4. Spatial Organization of Regulatory Elements

**FIGURE 4:**
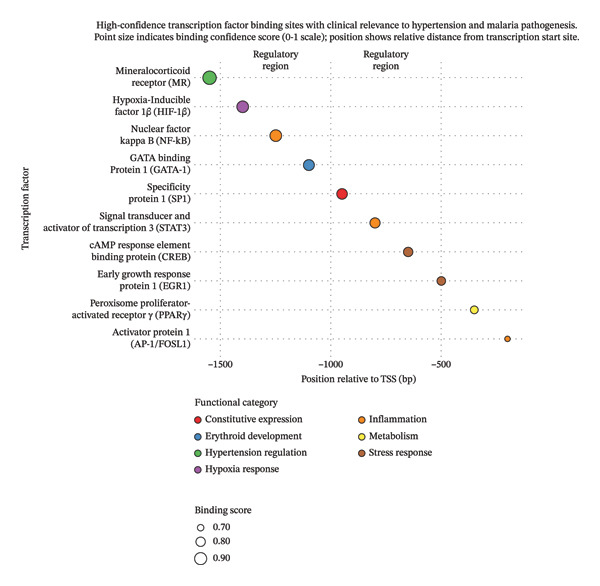
Spatial distribution of TF binding sites for *Rac1* promoter.

Spatial distribution analysis revealed nonrandom clustering of TFBSs, with significant enrichment in the −1500 to −500 bp region (Figure 4). This organizational pattern indicates several regulatory features: dense transcription factor binding clusters may function as enhancer elements coordinating *Rac1* expression; the physical proximity of MR, HIF, and inflammatory response elements suggests potential for integrated regulation by multiple signaling pathways; and asymmetric distribution of forward and reverse strand binding sites indicates sophisticated directional regulation of transcription initiation. This spatial organization supports a model of coordinated regulatory input integration that could enable context‐dependent *Rac1* expression in response to diverse physiological challenges.

## 4. Discussion

Our *in silico* findings suggest a potential mechanism for *Rac1*‐mediated bypass of RAAS blockade in antihypertensive therapy resistance. The presence of multiple MR response elements within the *Rac1* promoter suggests direct MR regulation, potentially explaining the observed therapy resistance through alternative pathway activation. In malaria‐endemic regions, where chronic inflammatory states are prevalent, *Rac1* upregulation via MR signaling could maintain hypertensive states despite conventional RAAS inhibition, creating a therapeutic challenge that has been clinically observed but mechanistically unexplained until now.

The GC‐rich nature of the *Rac1* promoter, coupled with multiple TSSs, indicates a robust, multilevel regulatory capacity that could sustain *Rac1* expression under therapeutic pressure. This complex promoter architecture may facilitate rapid adaptation to pharmacological challenges, contributing to the observed antihypertensive therapy resistance in malaria‐endemic populations. The promoter’s structural complexity, particularly its GC‐rich nature, may reflect evolutionary pressures such as historical malaria endemicity, which could have selected for genetic variants fine‐tuned inflammatory and erythrocytic responses via *Rac1* regulation.

Simultaneously, our identification of erythroid‐specific TFBSs and hypoxia‐responsive elements in the *Rac1* promoter provides crucial insight into enhanced malaria susceptibility. Based on predicted transcription factor binding, *Rac1*‐mediated cytoskeletal remodeling in erythrocytes could potentially facilitate *Plasmodium falciparum* invasion by modifying host cell membrane properties. This molecular vulnerability may be particularly consequential in populations where both hypertension and malaria coexist.

Furthermore, the inflammatory response elements identified in the *Rac1* promoter suggest its involvement in the delicate balance between protective and pathological immune responses to malaria infection. In individuals receiving RAAS inhibitors, which may inadvertently reduce innate antimalarial defenses, *Rac1* overexpression could create a permissive environment for parasite establishment and progression. This predicts a concerning therapeutic paradox where antihypertensive treatment could potentially exacerbate malaria susceptibility through *Rac1*‐mediated pathways.

The dense CpG island architecture, comprising 164 sites, identifies significant potential for epigenetic regulation that could underlie population‐specific effects observed in both antihypertensive response and malaria susceptibility. Genetic variants in the *Rac1* promoter region, particularly those affecting TFBSs or CpG dinucleotides, could alter regulatory dynamics and contribute to the geographical variations in disease presentation and treatment response. This epigenetic landscape suggests a potential molecular basis for the population‐specific patterns that have long been clinically observed but poorly understood.

These findings suggest several promising therapeutic strategies that could address this dual‐disease challenge. Combined RAAS and MR blockade may overcome *Rac1*‐mediated therapy resistance in malaria‐endemic regions, while context‐specific or tissue‐targeted *Rac1* modulation could potentially address hypertension and reduce malaria susceptibility through complementary mechanisms. Given the pleiotropic role of *Rac1* in constitutive cellular processes, systemic inhibition may carry significant toxicity and requires careful consideration. Epigenetic modulators that specifically target *Rac1* promoter methylation patterns offer the potential for population‐specific therapeutic approaches, acknowledging the genetic diversity that characterizes different endemic regions. Finally, strategic dual‐therapy protocols that combine optimized antihypertensives with antimalarial drugs, informed by individual *Rac1* regulatory status, could present a personalized medicine approach to this complex comorbidity.

These computational predictions require experimental validation (e.g., ChIP‐seq for TF binding, methylation‐specific PCR/reporter assays for promoter activity, and population genetics studies for variant analysis).

Notwithstanding, the convergence of these predicted regulatory mechanisms in the *Rac1* promoter offers a unified hypothetical framework for understanding the intersection of antihypertensive therapy resistance and malaria susceptibility. This integrated perspective highlights the importance of considering both infectious and noncommunicable diseases in therapeutic development, particularly in regions where these conditions frequently coexist and potentially interact through shared molecular pathways such as *Rac1* regulation.

## 5. Conclusion

The *Rac1* promoter exhibits a complex regulatory architecture that suggests a potential mechanism for MR‐mediated bypass of RAAS blockade, particularly relevant to angiotensin‐converting enzyme inhibitor (ACEI)/ Angiotensin II receptor blocker (ARB) resistance, while also predicting a link to erythrocyte remodeling pathways relevant to malaria pathogenesis. Its GC‐rich composition, which may reflect evolutionary adaptation to historical malaria endemicity, along with its diverse TFBSs and dense CpG distribution, offers a predictive molecular framework for understanding the observed antihypertensive therapy resistance and enhanced malaria susceptibility in endemic regions. These findings establish a hypothesis‐generating foundation for this clinically significant comorbidity in Sub‐Saharan Africa. Future therapeutic strategies targeting *Rac1* should prioritize context‐specific or tissue‐selective modulation to avoid potential toxicity arising from its pleiotropic physiological roles. This knowledge provides a roadmap for experimental validation and the development of dual‐disease therapeutic approaches tailored to high‐burden populations.

## Funding

Building a New Generation of Academics in Africa (BANGA‐Africa).

## Conflicts of Interest

The authors declare no conflicts of interest.

## Data Availability

The data that support the findings of this study are available from the first author, amekeselouis@gmail.com, upon reasonable request.
